# Molecular Transport and Growth of Lipid Vesicles Exposed to Antimicrobial Peptides

**DOI:** 10.1021/acs.langmuir.1c02736

**Published:** 2021-12-13

**Authors:** Josefine Eilsø Nielsen, Reidar Lund

**Affiliations:** Department of Chemistry, University of Oslo, Postboks 1033 Blindern, Oslo 0315, Norway

## Abstract

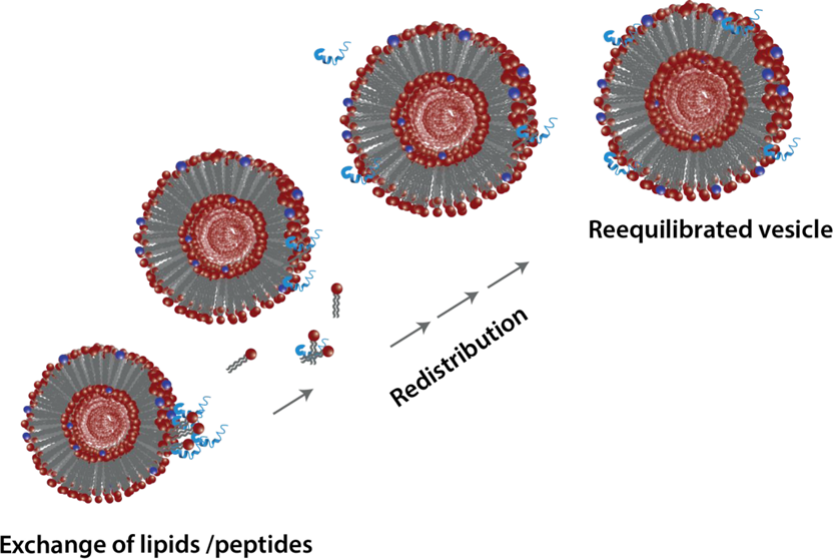

It is well-known that lipids constituting the cytoplasmic membrane undergo continuous reorganization to maintain the appropriate composition important for the integrity of the cell. The transport of lipids is controlled by mainly membrane proteins, but also spontaneous lipid transport between leaflets, lipid “flip–flop”, occurs. These processes do not only occur spontaneously under equilibrium, but also promote structural rearrangements, morphological transitions, and growth processes. It has previously been shown that intravesicular lipid “flip–flop” and intervesicular lipid exchange under equilibrium can be deduced indirectly from contrast variation time-resolved small-angle neutron scattering (TR-SANS) where the molecules are “tagged” using hydrogen/deuterium (H/D) substitution. In this work, we show that this technique can be extended to simultaneously detect changes in the growth and the lipid “flip–flop” and exchange rates induced by a peptide additive on lipid vesicles consisting of DMPC (1,2-dimyristoyl-*sn*-glycero-3-phosphocholine), d-DMPC (1,2-dimyristoyl-*d*_54_-*sn*-glycero-3-phosphocholine), DMPG (1,2-dimyristoyl-*sn*-glycero-3-phospho-(1′-*rac*-glycerol)), and small amounts of DMPE-PEG (1,2-dimyristoyl-*sn*-glycero-3-phosphoethanolamine-*N*-[methoxy(polyethylene glycol)-2000]). Changes in the overall size were independently monitored using dynamic light scattering (DLS). We find that the antimicrobial peptide, indolicidin, accelerates lipid transport and additionally induces limited vesicular growth. Moreover, in TR-SANS experiments using partially labeled lipid mixtures to separately study the kinetics of the lipid components, we show that, whereas peptide addition affects both lipids similarly, DMPG exhibits faster kinetics. We find that vesicular growth is mainly associated with peptide-mediated lipid reorganization that only slightly affects the overall exchange kinetics. This is confirmed by a TR-SANS experiment of vesicles preincubated with peptide showing that after pre-equilibration the kinetics are only slightly slower.

## Introduction

The cell membrane relies on controlled transport through the membrane to maintain its integrity, because an exact composition in terms of lipid and ions (protons, sodium, calcium, etc.) is required for healthy cell function. The balance is mainly kept by transmembrane proteins, which accurately regulate the composition of lipids and the balance of ions.^1^ The cytoplasmic membrane of eukaryotic and prokaryotic cells requires maintaining an asymmetric lipid composition on both the inner and the outer leaflets to function. In contrast to in-plane diffusion, it has long been known that lipid “flip–flop” is relatively slow (minutes–hours–days–months) in the absence of transmembrane proteins (“scramblases”, “flippases” and “floppases”),^2^ which have been found to significantly accelerate the process (seconds).^1,3,4^ Flippases and floppases are adenosine triphosphate (ATP)-dependent membrane proteins, as opposed to ATP-independent scramblases, which all move lipids to the inner monolayer and outer monolayer, respectively,^5^ and in that manner carefully maintain the lipid composition and rejuvenate the outer leaflet as lipids are synthesized within the cytoplasm. In the absence of these proteins, the lipid composition is thus rather constant, and if spontaneous “flip–flop” occurs in an uncontrolled manner, the lipid composition may be altered leading to destabilization of the membrane. Lipid scrambling and malfunction of membrane proteins have recently been related to human diseases including cancer, highlighting the importance of lipid dynamics.^1^ Destabilization of the bacterial membrane through accelerated lipid “flip–flop” has further been suggested as an essential step in the mode of action of antimicrobial peptides (AMPs).^6−13^

Lipid vesicles (liposomes) are often metastable where larger vesicles are usually more energetically favored than small ones due to the unfavorable curvature and strain of the latter. At the same time, thermal fluctuations of large vesicles lead to membrane budding and fission processes that give rise to the formation of small vesicles. Such processes are essential for cell signaling in multicellular organisms where small vesicles (exosomes) transfer important compounds (for example, RNA and various membrane and cytoplasmic proteins) between cells. The uptake of vesicles is affected by the spontaneous curvature and lipid composition and thus the ability of the leaflets to dynamically adjust their composition, that is, through lipid “flip–flop”.^14^ The growth mechanism of lipid vesicles is controlled by both fusion/fission and lipid exchange processes, directly determining the stability. Additives may alter the kinetic stability, either by enhancing fusion processes (divalent ions) and/or by increasing the solubility of the lipids leading to the Ostwald ripening mechanism where larger vesicles grow at the expense of the smaller ones due to asymmetric exchange.^15^

Time-resolved small-angle X-ray/neutron scattering (TR-SAXS/SANS) techniques have emerged as increasingly powerful tools to study nanostructures in the 1–100 nm range, with temporal resolution starting from a few milliseconds.^16^ The technique has been extensively used to study the self-assembly and morphology of soft matter systems.^17−19^ To probe lipid dynamics, it is essential to avoid perturbations from equilibrium, and it is desirable to monitor the nanostructure and potential changes simultaneously. Over the past decade, a novel hydrogen/deuterium (H/D) contrast variation technique based on TR-SANS as a “label-free” method has emerged to study molecular exchange processes.^19−21^ Contrary to other methods such as EPR, fluorescence, and temperature-jump experiments, the kinetic zero-average contrast (KZAC) TR-SANS method does not require chemical labeling or perturbation that disturbs equilibrium other than simple H/D exchange, thus avoiding significant alteration of the physicochemical properties of the system. The idea was originally developed to investigate the dynamics of block copolymer micelles,^20,21^ which was shown to be dominated by activated diffusion of single chains, a process that strongly depends on the surface tension between the solvent and the hydrophobic part, chain-length, and temperature.^20−23^ The TR-SANS method was later adapted to study lipid exchange in unilamellar vesicles (ULVs), that is, liposomes.^24−26^ As was first shown by Nakano and co-workers, both lipid “flip–flop” and intermembrane exchange can be deduced by monitoring the loss of SANS intensity over time.^24^ However, to derive the rate constants for lipid “flip–flop” and interbilayer exchange, a kinetic analysis was developed to resolve the net change of the integral intensity over time, and not by analyzing the (time-dependent) scattering curves. Thus, this experiment and similar later approaches^13,26,27^ did not take full advantage of the intrinsic spatial resolution of the SANS technique, and the determination of the “flip–flop” rates can only be deduced indirectly by analyzing the decay of the overall scattered intensity. Moreover, by analyzing only the net intensity, we cannot easily decipher potential parallel kinetic processes, such as vesicle growth or morphological transitions. Perez-Salas and co-workers later introduced an alternative approach where they used a form factor model with a time-dependent contrast to extract the exchange and “flip–flop” rates from the full scattering curves.^28,29^ The same approach was previously applied to polymer micelles^19,23,30^ and fully exploits both the spatial and the temporal resolution of SANS. In this work, we further expand the use of this type of analysis to simultaneous detect the structural evolution and spontaneous lipid transport of the vesicles, that is, lipid exchange and “flip–flop”.

The kinetics of lipid “flip–flop” can be determined by using asymmetric bilayers where one leaflet is selectively labeled. Conboy and co-workers used sum-frequency vibrational spectroscopy that requires deposition of a deuterated leaflet on a solid substrate (supported lipid bilayer). Upon “flip–flop”, the composition of the inner and outer leaflets is mixed, which can be followed by monitoring the amount of −CH_3_ (as opposed to −CD_3_) groups on the surface.^9,31,32^ The same idea has also been used in neutron reflectometry by Gerelli and co-workers who deposited a H/D labeled bilayer on silica and studied the loss in contrast over time. However, here it was found that interbilayer exchange was rate limiting and lipid “flip–flop” was too fast within the experimental time window.^33,34^ “Flip–flop” can also be detected by TR-SANS using asymmetric vesicles where one leaflet contains a deuterated lipid.^10^ Similar to the KZAC TR-SANS technique, “flip–flop” can then be monitored by the loss in the overall intensity, which in this contrast condition is not sensitive to intervesicular exchange processes. Using this approach, the authors investigate the effect of peptide insertion and find that the rate for “flip–flop” is accelerated. Several other studies have indicated that antimicrobial peptides (AMPs) induce changes in lipid dynamics, more specifically by accelerating “flip–flop” motion.^7,8,35,36^ At least in model systems, peptides can accelerate lipid “flip–flop” motions in a manner proportional to the amount of peptide inserted into the membrane.^12,37^ In one study, AMPs were found to induce “flip–flop” at concentrations much lower than those needed to cause the leakage of calcein.^8^ Hence, although there is significant evidence that AMPs may accelerate “flip–flop”, the molecular mechanism and the implications are not clear. Moreover, other mechanisms for peptide-induced lipid transport and redistribution need to be considered. In this context, interbilayer exchange may play an important role as this leads to redistribution of the lipid composition, first at the outer leaflet leading to a scrambling of the composition in the presence of other lipid sources.

In this work, we investigate peptide-induced vesicular growth as well as lipid exchange and “flip–flop” dynamics using the KZAC TR-SANS method combined with dynamic light scattering (DLS). We make use of both the structural and the temporal resolution of SANS by analyzing the full *Q*-range scattering curves. The approach we present is similar to what has been previously presented by Perez-Salas and co-workers.^28,29^ However, because we add an antimicrobial peptide to our vesicle system, we have to take into account a potential time-dependent change in the form factor of the vesicles caused by the peptide interaction, as well as changes in contrast resulting from lipid dynamics. Our results reveal that, upon addition of AMPs, the lipid dynamics, both the interbilayer exchange and the intrabilayer “flip–flop” motions, is considerably accelerated. For the “flip–flop”, the effect can primarily be attributed to a reduction of the activation energy of about 15% in addition to a reduction in the entropic barrier. This likely results from mediation of the headgroup–tail interaction and possibly complexation by the peptides. The analysis also shows that the acceleration of the lipid dynamics is accompanied by growth and, consequently, broader distribution of the vesicles. We also speculate that the change in dynamics may cause effects such as lipid scrambling and enhanced transport of solutes over the membrane that are detrimental to living bacteria. This thus may have implications for the mode of action of AMPs.

## Materials and Methods

### Materials

Synthetic DMPC (1,2-dimyristoyl-*sn*-glycero-3-phosphocholine), d-DMPC (1,2-dimyristoyl-*d*_54_-*sn*-glycero-3-phosphocholine), DMPG (1,2-dimyristoyl-*sn*-glycero-3-phospho-(1′-*rac*-glycerol)), d-DMPG (1,2-dimyristoyl-*d*_54_-*sn*-glycero-3-phospho-(1′-*rac*-glycerol)), and DMPE-PEG (1,2-dimyristoyl-*sn*-glycero-3-phosphoethanolamine-*N*-[methoxy(polyethylene glycol)-2000]) were purchased from Avanti Polar Lipids. The peptide indolicidin was purchased from Isca Biochemicals Limited. The Tris buffer was prepared by mixing 50 mM Tris-base with Tris-HCl (Sigma-Aldrich) in the correct ratio to achieve a pH of 7.4 in 50% D_2_O (Sigma-Aldrich) and 50% H_2_O (MilliQ).

### Sample Preparation

The lipids in a ratio of 75 mol % DMPC, 22.5 mol % DMPG, and 2.5 mol % DMPE-PEG (PEGylated lipids are added to stabilize the system against phase separation upon peptide addition as was previously described in Nielsen et al.^38^) were dissolved in a 1:3 methanol:chloroform solution. The organic solvents were removed completely under vacuum using a Heidolph rotary evaporator with a Vacuubrand vacuum pump. The resulting lipid film was hydrated with Tris buffer for at least 1 h at a temperature of 34 °C. After sonication for 15 min, the lipid dispersions were extruded through a 100 nm pore diameter polycarbonate filter (>21 times) using an Avanti mini-extruder fitted with two 1 mL airtight syringes. Indolicidin was dissolved in Tris buffer to the desired concentration.

### TR-SANS Data Collection

All SANS data were collected at the KWS1 SANS beamline, at the Heinz Maier-Leibnitz (FRM II) center, MLZ in Garching, Germany, except for the SANS data presented in Figure S3, which were collected at the D22 beamline, at the Institute Laue Langevin (ILL) in Grenoble, France. The D-liposomes were mixed with the H-liposomes (1:1) directly before the first measurement using a Finntip micropipette and mixed with either pure buffer (to make sure the concentration of the nonpeptide samples compares to that of the peptide samples) or peptide solution 1:1. The samples were filled into round Hellma quarts banjo-cells with a path length of 1 mm and kept in a temperature-controlled rack during the experiment.

### Extraction of Relaxation Function

The TR-SANS data can be evaluated by determining the relaxation function *R*(*t*) according to
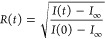
1where *I*(*t*) = ∫*I*(*Q*,*t*) d*Q* is the integral intensity at a given time, *I*_*∞*_ is the intensity of the premixed blend (a blend was prepared for each system by mixing the D- and H-lipids in the correct ratio in powder form, and preparing the liposomes as described above; in the case of the controls for the peptide samples, blends were mixed with indolicidin in a manner similar to that of the kinetic sample) representing the final state, and *I*(0) is the averaged intensity of the H-vesicles and D-vesicles measured separately representing the initial state before exchange and “flip–flop” has taken place.

#### Data Modeling of TR-SANS Data

**Figure 1:**
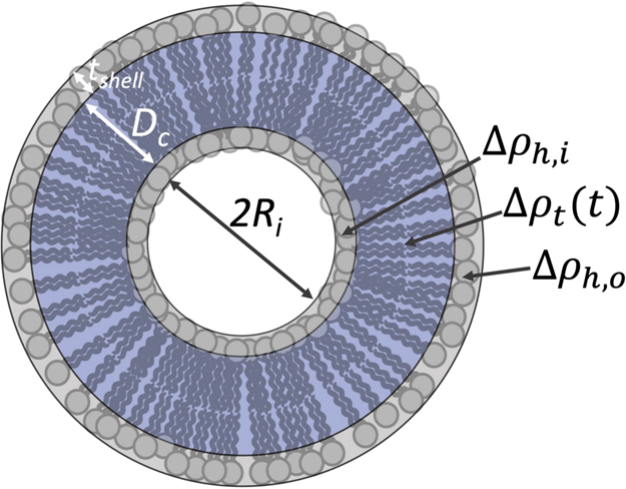
Schematic illustration of the concentric shell model.

For analysis of the TR-SANS data, a model of concentric shells of finite thicknesses was chosen (see Figure 1). Because of the restricted *Q*-range, the contrast of the vesicular hydrophobic core is characterized by a single time-dependent fraction, *f*_kin_(*t*), of deuterated/protiated lipids, and the bilayer was therefore divided into three concentric shells: one inner solvated shell consisting of headgroups and water with amplitude *A*(*q*)_h,i_, one middle shell of the tail groups with amplitude *A*(*q*)_t_, and one outer shell of headgroups and water with amplitude *A*(*q*)_h,o_. In our experimental design, we have used a ∼50% mixture of lipids with deuterated and protiated tails, respectively; however, the head groups are the same in all cases. We therefore have to consider that the contrast for the tail region of the inner and outer leaflets, Δρ(*t*)_t_, depends on time. The total form factor can thereby be expressed as follows:
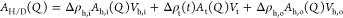
2where *V*_h,i_, *V*_t_, and *V*_h,o_ are the volumes of the inner headgroup, tail group, and outer headgroup shells, respectively. The volume and amplitude of each shell are defined as:
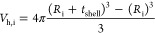
3
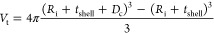
4
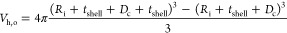
5
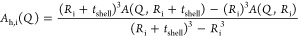
6
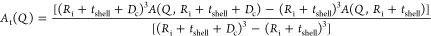
7
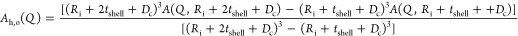
8where *R*_i_ is the inner radius of the vesicle, *D*_c_ is the total thickness of the hydrocarbon region, and *t*_shell_ is the thickness of each headgroup shell. The scattering contrast toward the lipid tails at the inner and outer leaflets depends on time and can be written as
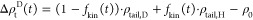
9
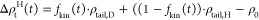
10Here, *f*_kin_(*t*) is the excess fraction of either H- and D-lipid in the bilayer.

To consider the hydration of the inner and outer shells, Δρ_h,i_ and Δρ_h,o_ are calculated as follows:
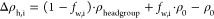
11
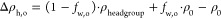
12where i is the inner and o is the outer headgroup, ρ_headgroup_ is the scattering length density of the lipid headgroup, and ρ_0_ is the scattering length density of the water. The fraction of water in the inner and outer shells,^39^*f*_w_, is given by
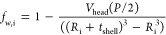
13
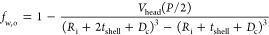
14where *P* is the aggregation number equal to the number of phospholipids in each vesicle.
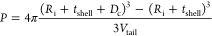
15where *V*_tail_ is the volume occupied by the hydrophobic tails of the phospholipid.

The scattering from the PEG chains was included in the fit model for SANS data assuming a Gaussian random coil confirmation on the inner and outer leaflets.^40,41^ The total intensity is then given by the following expression:
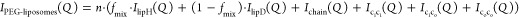
16where *n* is defined as
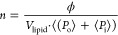
17where ϕ is the volume fraction and *V*_lipid_ is the total volume of the phospholipid taken as the average between weighted DMPC and DMPG. The average aggregation number and intensities were calculated assuming a Gaussian distribution, *g*(*R*_in_), of the inner radius of the vesicles.
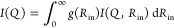
18Also, *I*_lipH/D_(*Q*) is the scattering intensity for the H- and D-liposomes calculated as
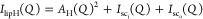
19
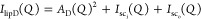
20where *A*(*Q*) is calculated according to eq 2 with the only difference in the H- and D-type liposomes given by the contrast to the tails (eqs 9 and 10), and *I*_sc_i__(*Q*) and *I*_sc_o__(*Q*) are the interference cross-terms of the outer and inner chains with the bilayer of the H-liposomes and D-liposomes (dependent on the *A*(*Q*)):
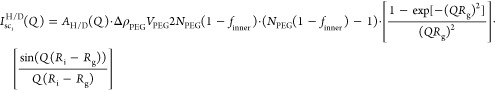
21
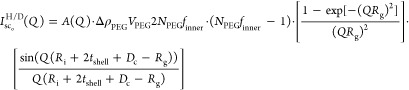
22In this expression, Δρ_PEG_ is the excess scattering length density, *V*_PEG_ is the partial specific molecular volume of a single PEG chain, *R*_g_ is the radius of gyration of the chains, *f*_inner_ is the fraction of PEG in the inner leaflet, and *N*_PEG_ is defined as the number of PEG chains per liposomes given by
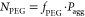
23*f*_PEG_ is the fraction of PEG-modified lipids in the liposomes, and *P*_agg_ = *P*_i_ + *P*_o_ is the aggregation number of the liposomes.

*I*_chain_(*Q*) is the scattering from the PEG chains alone given by
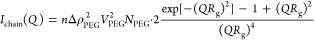
24The last terms, *I*_c_i_c_i__(*q*) and *I*_c_o_c_o__(*q*), are the interference terms between the PEG chains attached to the inner surface of the vesicles and between the PEG chains on the outer surface, respectively, while *I*_c_i_c_o__(*q*) is the interference between the inner and outer PEG chains:
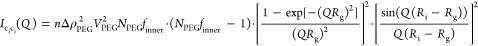
25
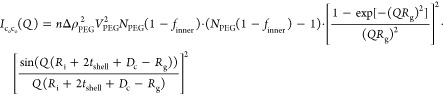
26
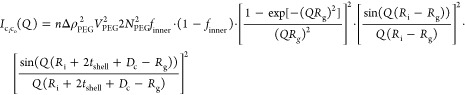
27

#### Calculation of Thermodynamical Parameters from TR-SANS Data

Following Nakano et al.,^24^ the lipid transport processes can be described by the following differential equations using the rate constants of exchange (*k*_ex_) and “flip–flop” (*k*_flip_):
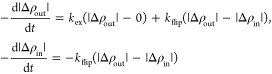
28where Δρ_out_ and Δρ_in_ are the contrast of the inner and outer leaflets of the vesicles with the solvent. As we have used a zero-average contrast solvent, the H- and D-vesicles can be assumed to have identical absolute values of contrast where one is positive and the other is negative.

With the initial condition that Δρ_out_(0) = Δρ_in_(0) = 1 and taking an average of |Δρ_out_| and |Δρ_in_|, the *R*(*t*), normalized contrast, has been explained by a double-exponential decay function:^24^
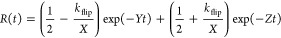
29where , , and .

To extract further thermodynamical parameters, ln *k*_ex_ and *k*_flip_ can be plotted against the inverse temperature in kelvin, 1/*T*, for samples measured at different temperatures giving an Arrhenius-type relationship. From this analysis, we obtain the activation energy, *E*_a_, and the fundamental time constant, τ_0_, according to
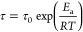
30where τ = 1/*k*, *R* is the universal gas constant, and τ_0_ is a system-specific constant and is related to the time between each time the molecule “attempts” to overcome the energetic barrier.^42^
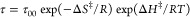
31where Δ*S*^‡^ is the entropy change, Δ*H*^‡^ is the enthalpy change, and τ_00_ is the estimated fundamental time constant related to eq 31 as τ_00_ = τ_0_·exp^–1^.^43^

### DLS Experiments

Dynamic light scattering experiments were performed using a DLS/SLS instrument equipped with a Cobolt high performance DPSS laser 100 mW (660 nm) from LS-instrument (Fribourg, Switzerland). The sample solutions were filtered in an atmosphere of filtered air through 5 μm filters (Millipore) directly into precleaned 2 mm NMR tubes. The concentration of liposomes was lowered to 0.5 mg/mL to avoid multiple scattering. Experiments at 0.25 mg/mL were included to check for concentration-dependent effects.

The correlation functions were analyzed using a single stretched exponential:
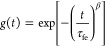
32where τ_fe_ is the effective relaxation time and β (0 < β ≤ 1) is a measure of the width of the distribution of relaxation times. Further, the mean relaxation time is given by
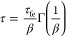
33where  is the gamma function of β^–1^. In the present work, the relaxation mode was observed to be diffusive in all cases (*q*^2^-dependent).

The hydrodynamic radius (*R*_h_) can be calculated through the Stokes–Einstein relationship from the relaxation time because the relaxation mode is diffusive:
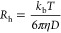
34where *T* is the temperature, η is the viscosity of the medium, *D* is the mutual diffusion coefficient (*D* = 1/τ*q*^2^), and *k*_b_ is the Boltzmann constant.

## Results and Discussion

**Figure 2:**
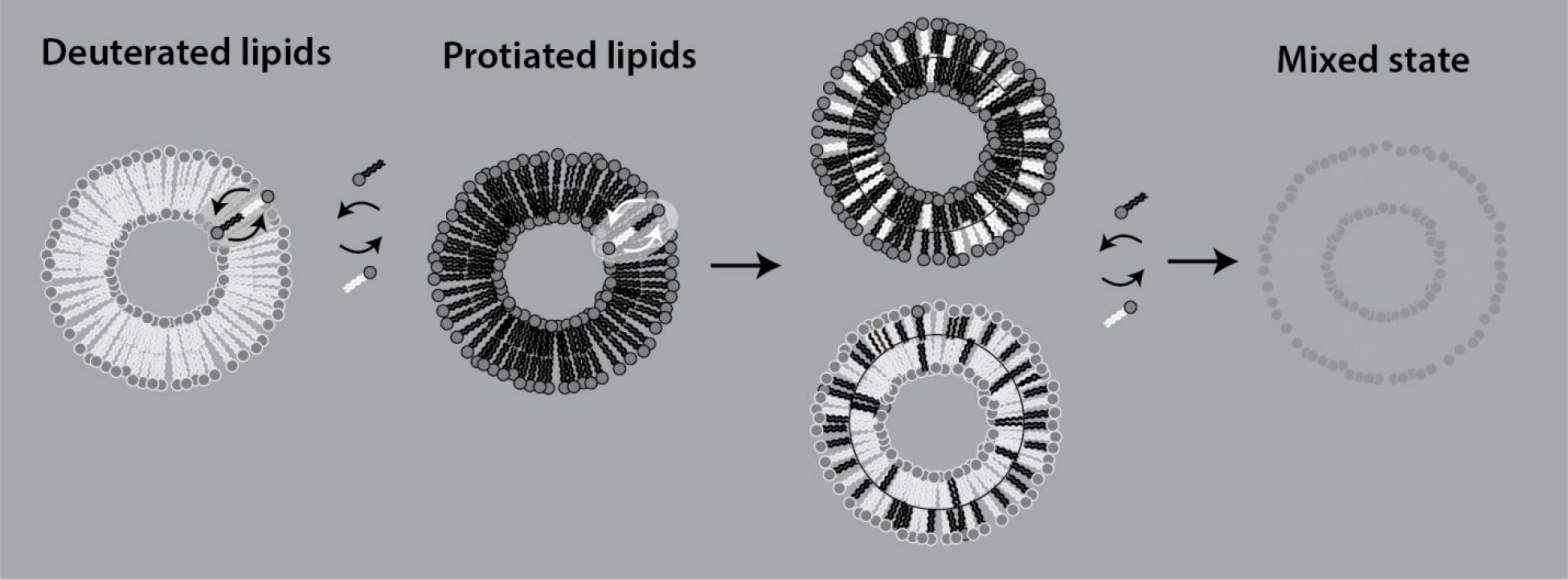
Schematic illustration of the TR-SANS technique designed to resolve the lipid dynamics, intravesicular “flip–flop”, and intervesicular exchange processes. The method, first developed for micelles,^20^ is based on mixing deuterated vesicles with protiated vesicles in a solvent that consists of about 50% H_2_O/D_2_O, matching the average scattering length density. As the molecules rearrange, contrast is lost and the neutron scattering intensity gradually decreases.

**Figure 3:**
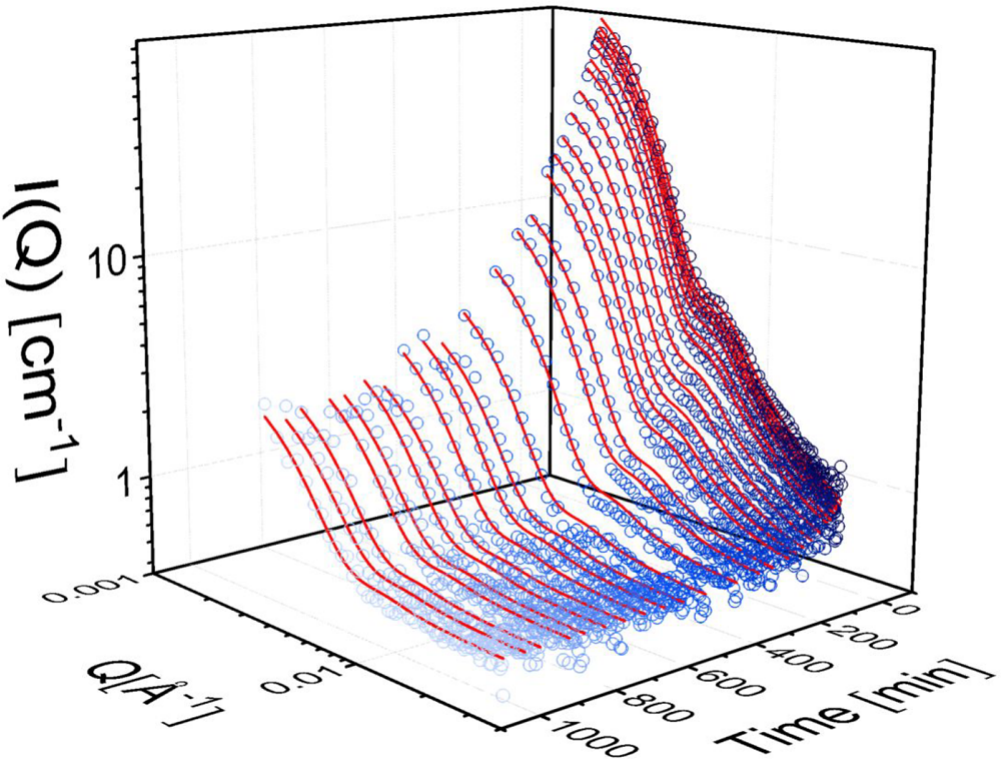
Scattered intensity plotted as a function of *Q* and the time obtained for 2.5 mg/mL DMPC/DMPG lipid vesicles in 50 mM Tris buffer at 37 C, together with the best fit. The last curve represents the premixed blend, *I*_*∞*_.

The TR-SANS method illustrated in Figure 2 is based on mixing protiated, H-labeled (black) and deuterated, D-labeled (white) vesicles and observing the decay in the scattering intensity over time. As the molecules mix and the average contrast decreases toward the mean solvent background (50% H_2_O/D_2_O solvent), the intensity decreases. An example of results obtained from using this method is given for liposomes at 37 °C in Figure 3, where the scattered intensity as a function of the *Q*-vector is plotted at different times after the solutions are mixed. The results reveal that, as expected, the intensity decreases gradually with time as the contrast is lost. However, the intensity is related to a change in contrast of both the inner and the outer leaflets, which are not necessarily following the same time dependence. Thus, first we developed a multishell model for vesicles where the time-dependent contrasts of the inner and outer leaflets are allowed to vary independently (see details in the Supporting Information and results from this approach in Figure S1). However, while the model was able to reproduce the data, the contrast between the leaflets proved not to be sufficient to extract unambiguous results (due to the restricted *Q*-range of the data). We thus proceeded to a simpler model where the contrast of the vesicular hydrophobic core is characterized by a single time-dependent fraction, *f*_kin_(*t*), of deuterated/protiated lipids. The fit results are shown as solid lines demonstrating an excellent description of the data. As can be observed at low *Q*, the data exhibit an upturn, that is, residual intensity, even at the near contrast matched conditions. This effect, which also is naturally described by our model, comes from the finite scattering contribution of the small amount of fully protiated PEG chains that still contributes coherently to the scattered intensity.

**Figure 4:**
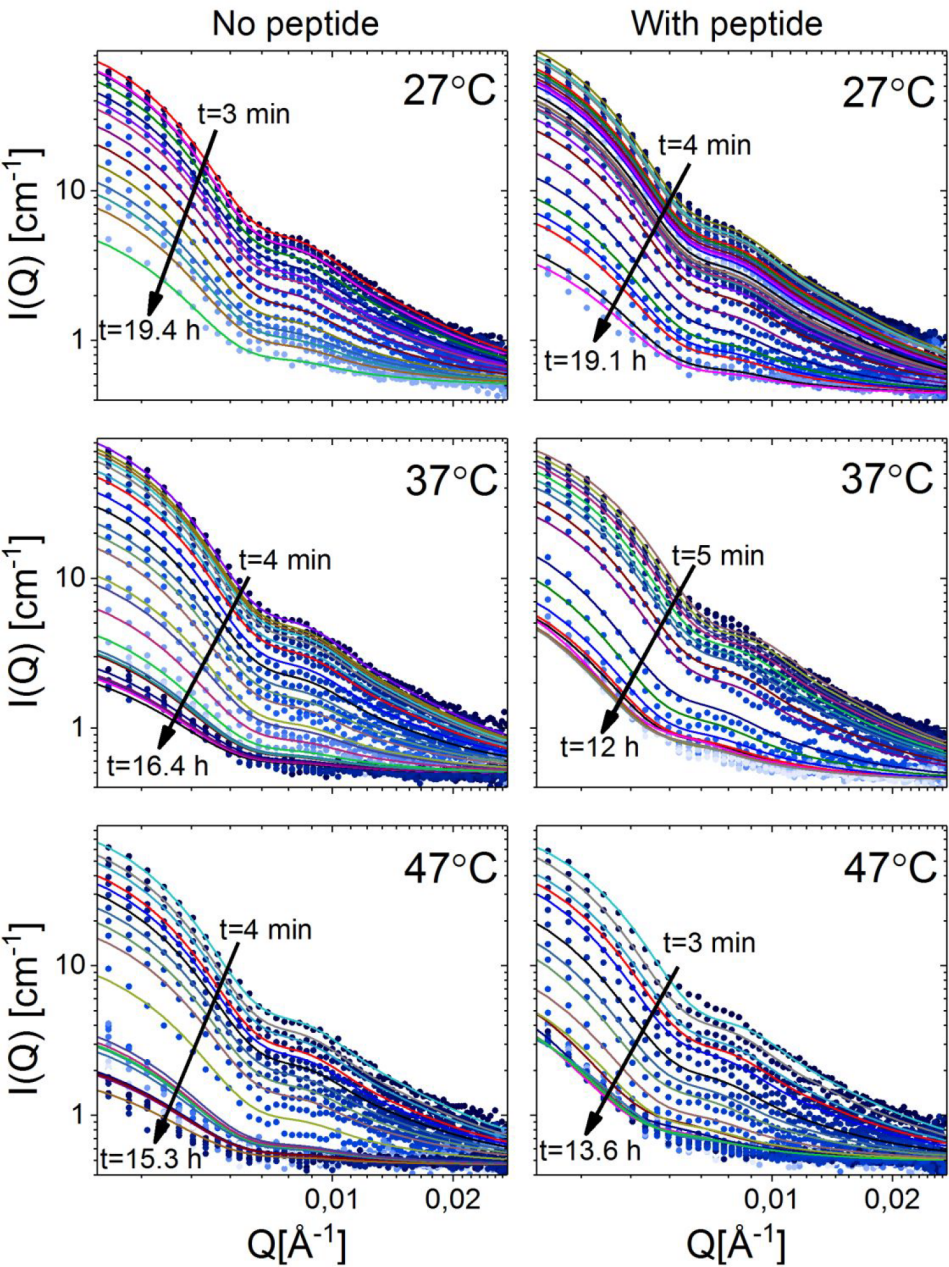
TR-SANS data including fits of DMPC/DMPG/DMPE-PEG vesicles with and without added peptides (1:20 indolicidin) at different temperatures. The start and end times are indicated. The last curve in each plot represents the premixed blend, *I*_*∞*_.

To evaluate the kinetic process in detail, we proceeded to perform experiments at various temperatures, 27, 37, 47, and 57 °C. The data are shown in Figure 4. As can be seen from the plots, the scattering model is able to explain the data of the pristine liposomes at the three lowest temperatures by only varying the contrast of the tail layer. However, unfortunately at 57 °C, the data statistics and time resolution are not sufficient to allow a full scattering curve analysis. The structural fit parameters giving information on the particle size and membrane thickness are presented in Table S1. The results from the fit analysis are consistent with prior published SAXS and SANS data on liposomes consisting of the same lipid mixtures.^38^

In nature, the rate of lipid “flip–flop” is highly regulated by membrane proteins, and it is known that the addition of drugs, for example, AMPs, may also affect the lipid motion. In these cases, it is especially interesting to use a methodology that is able to determine both the exchange and “flip–flop” rate as well as any other kinetic processes such as changes in the morphology or the size of the vesicles as this may be an important factor in fully understanding how the addition of peptides or proteins affects lipid membranes. We thus subsequently added a peptide, indolicidin, which is known from our previous studies to insert into the outer leaflet of the membrane^38^ and accelerate the lipid transport.^13^ The TR-SANS results on liposomes with added 1:20 (peptide:lipid ratio) indolicidin at 27, 37, and 47 °C are shown together with the best fit in Figure 4. As seen from the plots, the model is able to fully explain the scattering data. It became apparent that, to satisfactorily describe the data, the size of the liposomes in addition to the contrast were set as free parameters (see Table S1). The thickness of the bilayer upon peptide addition was also initially set to vary in the fit analysis; however, the results revealed that this parameter remained constant, which is supported by previously published SAXS and neutron reflectometry (NR) data.^38,44^

**Figure 5:**
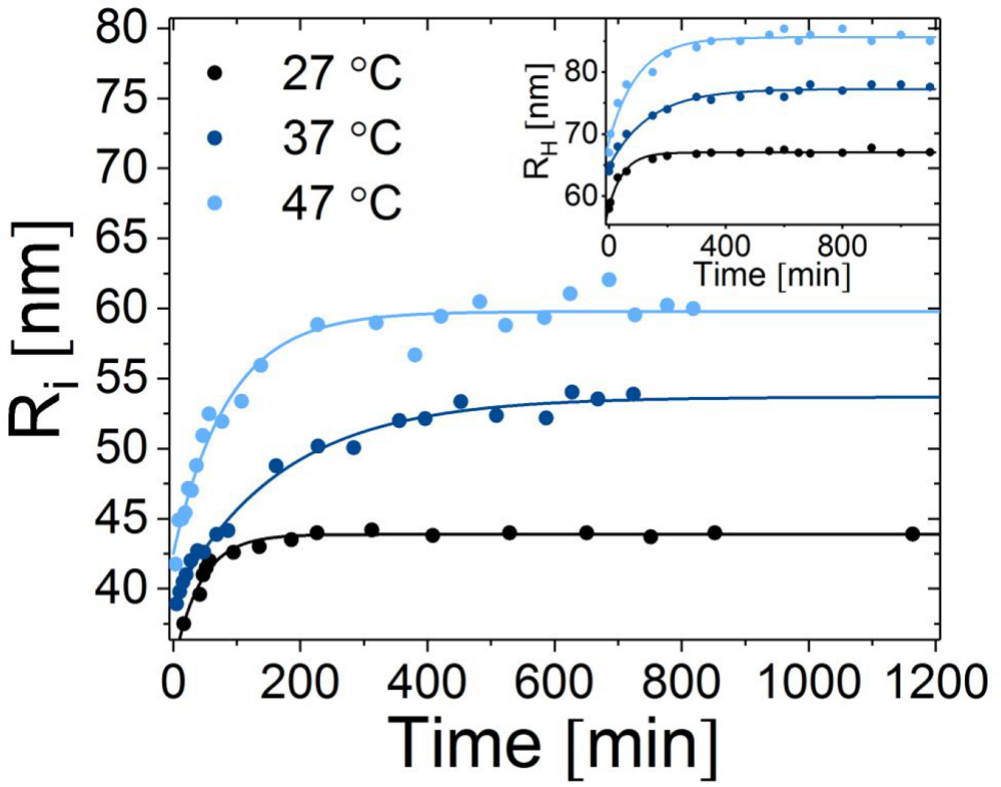
Inner radius of the liposomes with 1:20 indolicidin, as a function of time based on the fit analysis of TR-SANS data. The inset shows the hydrodynamic radius of liposomes with 1:20 indolicidin at the same temperatures obtained from DLS.

The inner radii (*R*_i_) of the liposomes as a function of time at different temperatures have been plotted in Figure 5. As seen, the size of the liposomes initially increases after peptide addition, but eventually reaches a stable plateau. The growth of the particles was found to follow the exponential expression , where Δ*R*_in/h_ is the difference between the end and start sizes of the particles, τ is the time constant, and *R*_start,in/h_ is the initial liposome size measured by TR-SANS (*R*_in_) or DLS (*R*_h_). When comparing the effect at different temperatures, it is obvious that the total growth is more pronounced at increasing temperatures. The same trend can be observed using DLS showing that the hydrodynamic radius (*R*_H_) also increases over time. This demonstrates that because both the inner vesicle radius and the hydrodynamic radius increase, the growth is related to an increase in average aggregation number of the lipids and not by simple insertion of peptides. Note that the samples for these experiments are not exactly the same as the samples used for the TR-SANS experiments, and therefore absolute values should not be compared directly. Our data also show that liposomes without added peptides are very stable over a long time period. We have previously tried to follow these samples over months without any observed changes in size or bilayer structure. The liposomes contain 2.5% PEGylated DMPE-lipids to increase the stability of the vesicles against self-aggregation in the presence of a cationic substrate such as indolicidin, as was previously described by Nielsen et al.^38^ The PEGylation together with the inclusion of 25% negative charged (DMPG and DMPE-PEG are both anionic) lipids provide an explanation of the significant physical stability observed in the DLS data for the pristine liposome system.

Upon peptide addition, the physical stability of the PEGylated liposomes is disturbed, causing growth of the particles. Generally, the growth can occur via different mechanisms, for example, fusion, or through molecular exchange where larger vesicles grow at the expense of the smaller ones.^45^ From previous SAXS and SANS results, we know that incorporation of 2.5% PEGylated DMPE is sufficient to stabilize the liposomes from aggregation and induction of multilamellar structures caused by peptides.^38^ Nevertheless, to investigate whether peptide-induced fusion of vesicles may occur, we performed DLS measurements at different peptide concentrations (0.25 and 0.5 mg/mL) (Figure S2). The data unequivocally show that the process is concentration-independent, and thus fusion seems not to play an important role. In addition, previously reported TR-SANS experiments by Nielsen et al. on the same system show no detectable change in lipid transport when the concentration is varied.^13^ This indicates that vesicle fusion events or collision-induced exchange processes do not frequently occur. Previously, NR and atomic force microscopy (AFM) experiments have revealed that the addition of indolicidin to supported lipid bilayers (SLBs) composed of DMPC and DMPG causes solubilization and removal of lipids, but only at large P:L values. We hypothesize that the addition of indolicidin leads to an inhomogeneous partial dissolution of a limited amount of lipids. This leads to a redistribution of small lipid/peptide mixed micellar structures that become available. Once the lipids/peptides have been homogeneously distributed, the vesicles stabilize into the new size distribution and metastable state. We should also note that the apparent time constant for vesicle growth is faster for 27 °C as compared to the higher temperatures. At the same time, the amplitude for growth, Δ*R*, is decreasing with decreasing temperature and is the lowest for 27 °C (Table 1). We interpret this as a consequence of less materials being solubilized at low temperature, therefore leading to faster redistribution.

**Table 1:** Exponential Growth Fit Parameters of Radii of Liposomes with Indolicidin as a Function of Time at Different Temperatures

	TR-SANS			DLS		
temp [°C]	*R*_start,in_ [nm]	Δ*R*_in_ [nm]	τ_SANS_ [min]	*R*_start,h_ [nm]	Δ*R*_h_ [nm]	τ_DLS_ [min]
27	34.5 ± 0.8	9.4 ± 0.8	42 ± 5	58.2 ± 0.3	8.8 ± 0.3	50 ± 5
37	39.2 ± 0.3	14.5 ± 0.4	169 ± 16	64.8 ± 0.4	12.5 ± 0.5	139 ± 15
47	42.5 ± 0.7	17.3 ± 0.7	87 ± 10	68.9 ± 0.8	16.8 ± 0.9	98 ± 15

It is interesting to note that a trial experiment (Figure S3) of vesicles preincubated with peptides shows slightly slower exchange/flip–flop processes, thus supporting the hypothesis that partial solubilization increases the kinetic rate, at least at the initial stage of the process. From the analysis, we obtain a faster *k*_ex_ for the freshly mixed peptide/lipid samples as compared to the preincubated sample (Table S2), while the difference in *k*_flip_ was less pronounced. However, more systematic TR-SANS studies are necessary to conclude any further.

**Figure 6:**
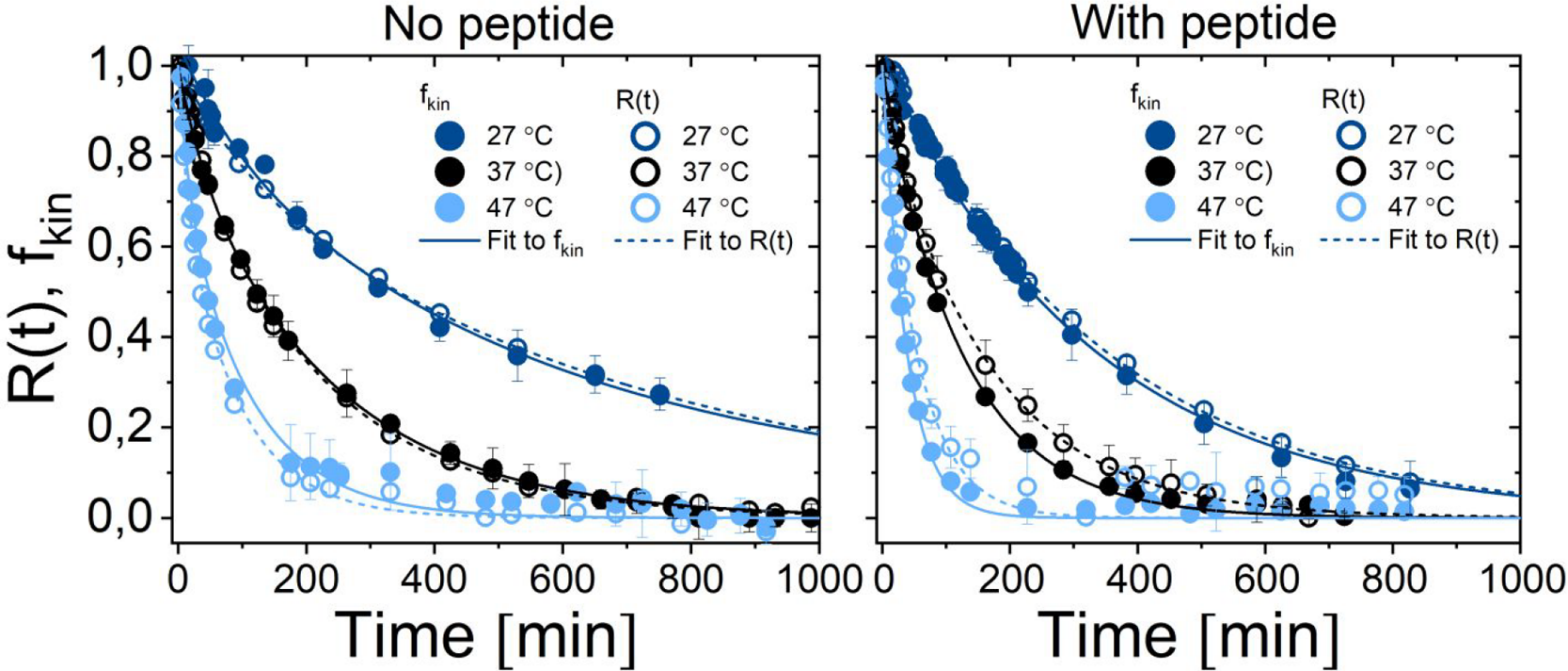
Results for the exchange and flip–flop of liposomes without peptide and with peptide at 27, 37, and 47 °C. The plot shows the excess fraction of either H- and D-lipid in the bilayer (*f*_kin_) based on direct modeling of the full *Q*-range TR-SANS data and the *R*(*t*) curves extracted from the integral net loss of scattering intensity (eq 1). To extract information on the exchange (*k*_ex_) and “flip–flop” (*k*_flip_) rates, the data have been analyzed using the model presented in eq 29.

Apart from the changes in particle size, we extract information on the excess fraction of either H- and D-lipid in the tail region as plotted in Figure 5. To compare the results from the direct and indirect approaches to analyze the TR-SANS data, the traditional *R*(*t*) curve (eq 1) has been plotted together with the *f*_kin_(*t*) parameter in Figure 6, and the data were analyzed using the expressions shown in eq 29 to extract the exchange (*k*_ex_) and “flip–flop” (*k*_flip_) rates. As seen from the results of the system with no added peptide, the *R*(*t*) and *f*_kin_(*t*) parameters are comparable, while in the system with added peptide the decay rate seems to be slightly higher when using the full the *Q*-range modeling approach (*f*_kin_(*t*)) rather than the integral intensity approach (*R*(*t*)). This can be explained by a simultaneous increase in the vesicle size of the liposomes upon peptide addition as described above, resulting in a change of the form factor, which is not taken into account in the integral intensity approach used to calculate the *R*(*t*) curve.

**Figure 7:**
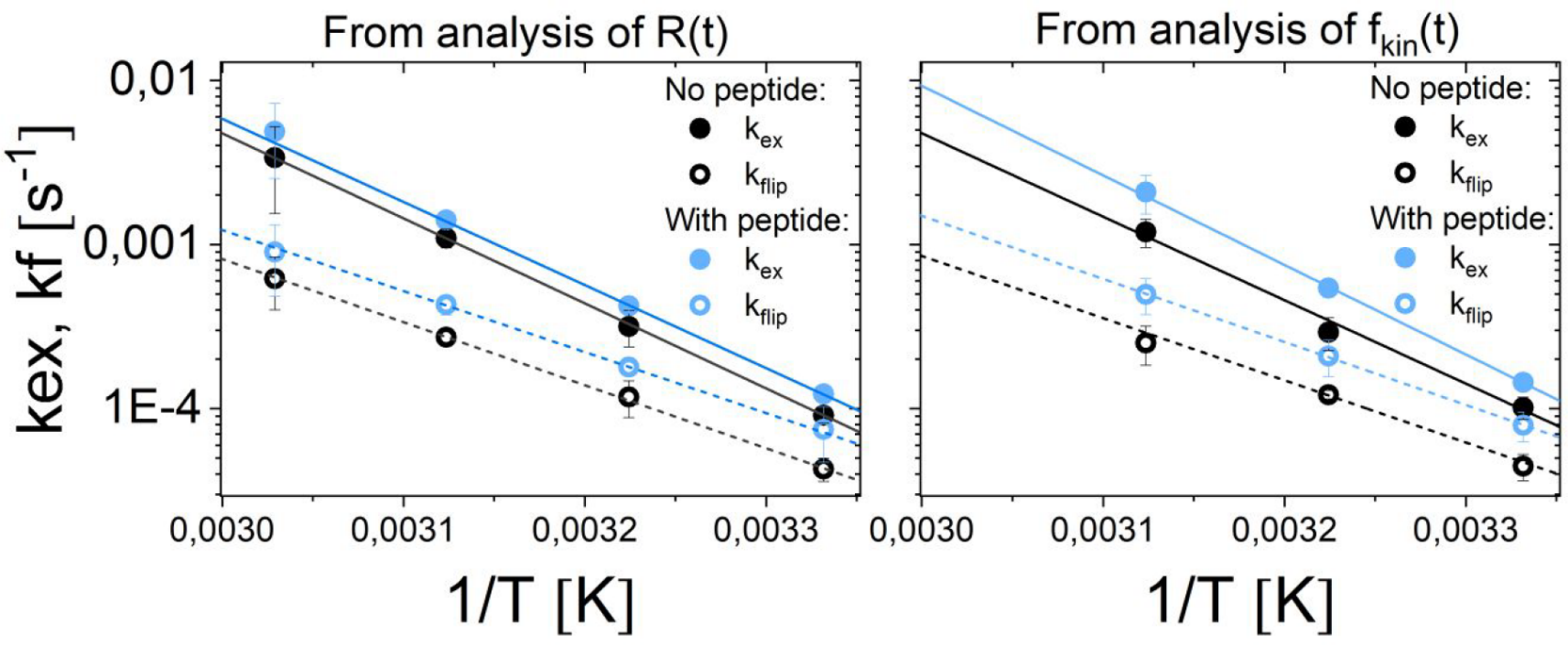
Arrhenius plots for liposomes with and without 1:20 indolicidin based on the fit of *R*(*t*) and *f*_kin_(*t*).

The exchange and “flip–flop” rates found from the three-shell model (*f*_kin_(*t*)) and the *R*(*t*) curve are shown in the Arrhenius plots in Figure 7. When comparing the parameters for the liposomes with and without peptides, we find the same overall trends as in Nielsen et al.^13^ where indolicidin slightly lowers the activation energy of the lipid “flip–flop” process. Interestingly, comparing the data in Table 2, we see that the entropy of activation, Δ*S*^⧧^, is also reduced. This may suggest that the lipids complex with the peptide, leading to a more ordered “activated state” that proceeds to flip through the membrane. As was previously reported, for a peptide with stronger interactions, LL-37, the activation energy of the flip–flop process remains constant, while the entropic barrier is significantly reduced.^13^ However, for the exchange process we observe that LL-37 causes a significant change in the enthalpic barrier. Thus, for both indolicidin and LL-37, we observe a decrease in Δ*S*^⧧^, which causes an acceleration of the flip–flop and exchange processes, suggesting that the peptide induces lipid ordering through, for example, transition complexes. Although the mechanism(s) are not clear, many other AMPs have been found to accelerate the lipid dynamics,^6−13,36,46^ warranting the need for a more detailed analysis of the microscopic pathway using, for example, computer simulations.

**Table 2:** Thermodynamical Parameters of Liposomes with and without Peptide

	lipid exchange					lipid “flip–flop”				
	*k*_ex_^a^ [10^–3^ min^–1^]	*E*_a_ [kJ/mol]	Δ*H*^⧧^ [kJ/mol]	*T*Δ*S*^⧧^ [kJ/mol]	Δ*G^⧧^* [kJ/mol]	*k*_flip_^a^ [10^–3^ min^–1^]	*E*_a_ [kJ/mol]	Δ*H*^⧧^ [kJ/mol]	*T*Δ*S*^⧧^ [kJ/mol]	Δ*G^⧧^* [kJ/mol]
No Peptide										
*f*_kin_(*t*)	18.4 ± 0.3	98 ± 9	95	0	97	7.3 ± 0.4	73 ± 7	70	–28	99
*R*(*t*)	18.9 ± 0.9	99 ± 1	96	1	97	7.1 ± 0.8	74 ± 1	71	–27	99
With Added Peptide										
*f*_kin_(*t*)	32.1 ± 0.7	104 ± 3	101	6	95	15.4 ± 1.4	68 ± 2	65	–32	97
*R*(*t*)	25.3 ± 1.1	98 ± 2	95	0	96	11.0 ± 0.8	66 ± 2	60	–38	97

a The rate constants (*k*) of exchange and “flip–flop” are extrapolated to 37.0 °C from the Arrhenius data.

The experiments presented so far were performed on lipid mixtures, and thus the parameters relate to both DMPC and DMPG. Consequently, the rate, and in particular the activation barriers, deviate from those previously obtained by Nakano et al. on pure DMPC vesicles.^24^ Homan and Pownall found that when comparing lipids with PG and PC headgroups the latter has a significantly slower flip–flop rate.^43^ In fact, Nakano et al. reported that the POPC kinetics^25^ was too slow to be efficiently observed, while POPG could be monitored using TR-SANS.^47^

**Figure 8:**
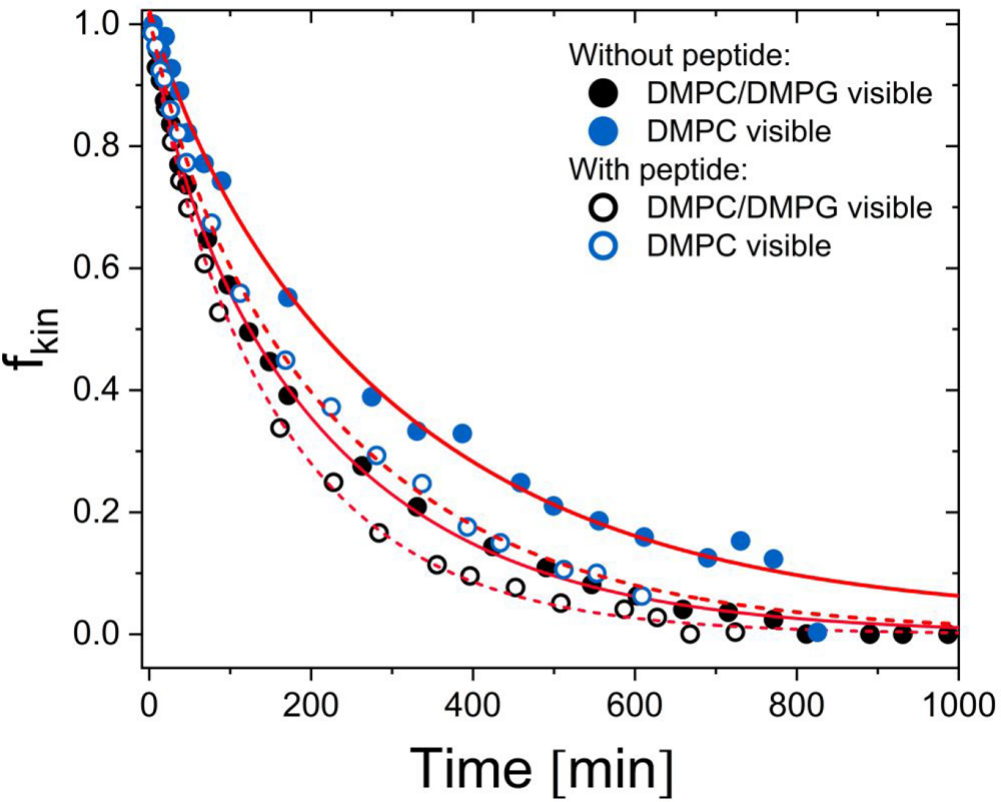
TR-SANS experiments where the kinetics of (i) both lipids are visible (d-DMPC–d-DMPG + h-DMPC–h-DMPG) or (ii) only the kinetics of DMPC can be isolated (d-DMPC–d-DMPG + h-DMPC–d-DMPG). The experiment was conducted at 37 °C and analyzed using the model outlined in the text.

To be able to differentiate and investigate the effect of the headgroup, we did a control experiment comparing liposomal systems where h-DMPC–h-DMPG vesicles were mixed with d54-DMPC–d54-DMPG and d54-DMPC–h-DMPG vesicles, respectively. In the first case, the kinetics of both DMPC and DMPG are observed simultaneously, while in the latter we are selectively able to monitor DMPC. The results from these experiments are shown in Figure 8.

The results presented in Figure 8 show that the kinetic curves corresponding to the DMPC-labeled sample decay significantly slower than those for the sample where both lipids are visible. For the peptide-free solution, we find *k*_ex_ 9.1 × 10^–3^ min^–1^ and *k*_flip_ 6.8 × 10^–3^ min^–1^ for the DMPC visible liposomes (Table S3), as compared to *k*_ex_ 1.8 × 10^–2^ min^–1^ and *k*_flip_ 7.3 × 10^–3^ min^–1^ for the “full contrast sample” where both DMPC/DMPG are visible. From the comparison, we see that DMPC flips and exchanges significantly slower than DMPG. This is in accordance with previous results from Homan and Pownall^43^ and Nakano et al.,^47^ which must be attributed to the nature of the head groups.

However, a detailed quantitative comparison of these data should be done with some caution because the difference in intensity between the start/end point of the d54-DMPC–h-DMPG system is much smaller than that for the d54-DMPC–d54-DMPG system. However, on the basis of the results, it seems likely that there indeed is a difference in the kinetics of DMPC and DMPG, where the first mentioned has significantly slower exchange and flip–flop rates than the latter. Although the slightly smaller head as well as the charge and counterion may play a role, we cannot conclude further, and most likely computer simulations are necessary for more detailed information about the mechanism. Nevertheless, it is clear that the addition of the AMP has a similar effect on both lipids.

## Conclusion

Lipid vesicles are frequently used as a model system for understanding the biophysical behavior of membrane systems. In this work, we have developed a scattering model that can be used to analyze full *Q*-range TR-SANS to investigate both lipid “flip–flop” exchange and vesicular growth simultaneously. We have demonstrated that the model is able to explain scattering data for pure lipid vesicle systems as well as liposomes in the presence of accelerating substrates like peptides or proteins. Upon analyzing TR-SANS data from liposomes with an added antimicrobial peptide, indolicidin, we found that a change in the size of the particles was necessary to fully explain the progression of the scattering curves over time. This peptide-induced growth can be explained by partial dissolution of lipids followed by a reorganization process that leads to vesicular growth into larger and more polydisperse vesicles. However, the process is transient, and as the peptide is presumably uniformly distributed over the vesicles, the system settles into a new equilibrium. By analyzing the time evolution of liposomes at various concentrations, we observe that both the vesicular growth rate and the exchange kinetics are independent of number density of vesicles, demonstrating that fusion/fission events do not play an important role. On a technical note, our results show that the variation of overall size interferes with the classical detection of flip–flop and exchange presented by Nakano and co-workers,^24^ and thus a full time-dependent fit analysis must be included when structural relaxations occur in parallel to molecular exchange processes. When comparing results from analyzing the same TR-SANS data using the two methods, we found comparable results in the neat liposomes. However, for liposomes exposed to peptides, where we observe also structural changes, we find slightly different decay rates. This is reflected in a slight change in the activation energy. We therefore conclude that a direct modeling approach is needed and also provides more information on the system, when a concurrent structural evolution is observed. However, this approach requires good statistics and is more time-consuming than the indirect approach. We furthermore observe using partially labeled lipid mixtures and TR-SANS that PG lipid dynamics is faster than PC dynamics. However, both are similarly affected by the peptide insertion. Also, Nguyen et al.^10^ found that alamethicin, melittin, and gramicidin all caused lipid scrambling in an asymmetric POPC/DMPC vesicle system, which practically remains stable over days in the absence of peptides. It is interesting to note that the magnitude of the effect on either lipid flip–flop and intervesicular exchange will depend on the exact peptide. In the present work, we see that indolicidin causes a reduction of both the entropic and the enthalpic contributions to the free energy of activation for the flip–flop process, whereas for the intervesicular exchange process, the entropic part is dominant. In contrast, LL-37, which was found to insert deeper into the membrane, shows the opposite trend; whereas for the exchange process the enthalpic activation energy and entropy are reduced, the effect is mainly entropic for the flip–flop process.^13^ Although the exact mechanism of how the AMP affects lipid dynamics on a molecular level is not entirely clear pending further investigation, our results provide comprehensive insight into the kinetic and thermodynamic contributions causing the acceleration of flip–flop and exchange in lipid model systems. We also shed significant light on the coupling of structural alterations, lipid dynamics, and the resulting growth kinetics of vesicles.

## Supporting Information Available

Four-concentric -shell model description; four-concentric-shell model results; structural fit parameter of liposomes using the three-shell model; DLS data on different liposome concentrations; TR-SANS data on preincubated liposome-peptide samples; and thermodynamical parameters comparing the DMPC and DMPG lipids (PDF)
