# Supplementary material for: Molecular Transport and Growth of Lipid Vesicles Exposed to Antimicrobial Peptides
Source: Langmuir. 2021 Dec 13;38(1):374–84. doi: 10.1021/acs.langmuir.1c02736 (PMC8757467; doi:10.1021/acs.langmuir.1c02736)
Supplement: Supplementary file 1 — la1c02736_si_001.pdf [file la1c02736_si_001.pdf]

Molecular Transport and Growth of Lipid Vesicles exposed to Antimicrobial Peptides

Authors: Josefine Eilsø Nielsen and Reidar Lund[1] *

Affiliations: [1]Department of Chemistry, University of Oslo, Postboks 1033 Blindern, 0315 Oslo, Norway.
*Correspondence to: reidar.lund@kjemi.uio.no

Four concentric shell model:

For a more direct extraction of k_{ex} and k_{flip} from TR-SANS data a model of four concentric shells of finite thicknesses was used. In this model the bilayer was divided into one inner solvated shell consisting of headgroups and water with amplitude $A(q)_{h,i}$, two middle shell of the tail groups with amplitude $A(q)_{t,i}$ and $A(q)_{t,o}$and one outer shell of headgroups and water with amplitude $A(q)_{h,o}$. In our experimental design, we have used a ≈ 50% mixture of lipids with deuterated and proteated tails respectively, however the head groups are the same in all cases. We therefore have to consider that the contrast for the tail region of inner and outer leaflet, $\Delta\rho(t)_{t,i}$ and $\Delta\rho(t)_{t,o}$depends on time. The total form factor of the can thereby be expressed as the following

$$A_{H/D}(Q) = \Delta\rho_{h,i}A_{h,i}(Q)V_{h,i} + \Delta\rho_{t,i}(t)A_{t,i}(Q)V_{t,i} + \Delta\rho_{t,o}(t)A_{t,o}(Q)V_{t,o} + \Delta\rho_{h,o}A_{h,o}(Q)V_{h,o} \tag{S1}$$

where $V_{h,i}, V_{t,i}, V_{t,o}, V_{h,o}$ are the volumes of the inner head- and tail group, and outer tail- and head group shells respectively. The volume of each shell is defined as:

$$V_{h,i} = 4\pi \frac{(R_i + t_{shell})^3 - (R_i)^3}{3} \tag{S2}$$

$$V_{t,\mathrm{i}} = 4\pi \frac{(R_\mathrm{i} + t_{shell} + D_c/2)^3 - (\mathrm{R_i} + t_{shell})^3}{3} \tag{S3}$$

$$V_{t,\mathrm{o}} = 4\pi \frac{(R_\mathrm{i} + t_{shell} + D_c)^3 - (\mathrm{R_i} + t_{shell} + D_c/2)^3}{3} \tag{S4}$$

$$V_{h,\mathrm{o}} = 4\pi \frac{(R_\mathrm{i} + t_{shell} + D_c + t_{shell})^3 - (\mathrm{R_i} + t_{shell} + D_c)^3}{3} \tag{S5}$$

$$A_{h,i}(\mathrm{Q}) = \frac{(R_i + t_{shell})^3 A(\mathrm{Q}, R_i + t_{shell}) - (R_i)^3 A(\mathrm{Q}, R_i)}{(R_i + t_{shell})^3 - R_i^3} \quad (S6)$$

$$A_{t,\mathrm{i}} = \frac{(R_\mathrm{i} + t_{shell} + D_c/2)^3 A(q, R_\mathrm{i} + t_{shell} + D_c/2) - (\mathrm{R_i} + t_{shell})^3 \mathrm{A}(\mathrm{q}, \mathrm{R_i} + t_{shell})}{(R_\mathrm{i} + t_{shell} + D_c/2)^3 - (\mathrm{R_i} + t_{shell})^3} \quad (S7)$$

$$A_{t,\mathrm{o}} = 4\pi \frac{(R_\mathrm{i} + t_{shell} + D_c)^3 - (\mathrm{R_i} + t_{shell} + D_c/2)^3}{3} \quad (S8)$$

$$A_{t,\mathrm{o}} = \frac{(R_\mathrm{i} + t_{shell} + D_c)^3 A(q, R_\mathrm{i} + t_{shell} + D_c) - (\mathrm{R_i} + t_{shell})^3 \mathrm{A}(\mathrm{q}, \mathrm{R_i} + t_{shell} + D_c/2)}{(R_\mathrm{i} + t_{shell} + D_c)^3 - (\mathrm{R_i} + t_{shell} + D_c/2)^3} \quad (S9)$$

$$A_{h,\mathrm{o}}(\mathrm{Q}) = \frac{(R_\mathrm{i} + 2t_{shell} + D_c)^3 A(\mathrm{Q}, R_\mathrm{i} + 2t_{shell} + D_c) - (\mathrm{R_i} + t_{shell} + D_c)^3 \mathrm{A}(\mathrm{Q}, \mathrm{R_i} + t_{shell} + +D_c)}{(R_\mathrm{i} + 2t_{shell} + D_c)^3 - (\mathrm{R_i} + t_{shell} + D_c)^3} \quad (S10)$$

where R_i is the inner radius of the vesicle, D_c is the total thickness of the hydrocarbon region, t_{shell} is the thickness of each head group shell. The scattering contrast towards the lipid tails at the inner and outer leaflets depend on time and can be written as:

$$\Delta\rho_{t,i}^{D}(\mathrm{t}) = (1 - f_{in}(t)) \cdot \rho_{tail,D} + f_{in}(t) \cdot \rho_{tail,H} - \rho_0 \quad (S11)$$

$$\Delta\rho_{t,o}^{D}(\mathrm{t}) = (1 - f_{out}(t)) \cdot \rho_{tail,D} + f_{out}(t) \cdot \rho_{tail,H} - \rho_0 \quad (S12)$$

$$\Delta\rho_{t,i}^{H}(\mathrm{t}) = f_{in}(t) \cdot \rho_{tail,D} + (1 - f_{in}(t) \cdot \rho_{tail,H} - \rho_0 \quad (S13)$$

$$\Delta\rho_{t,o}^{H}(\mathrm{t}) = f_{out}(t) \cdot \rho_{tail,D} + (1 - f_{out}(t) \cdot \rho_{tail,H} - \rho_0 \quad (S14)$$

Here $f_{in}(t)$ and $f_{out}(t)$ is excess fraction of either H- and D-lipid in the inner and outer leaflet respectively.

To consider the hydration of the inner and outer shell, $\Delta\rho_{h,i}$ and $\Delta\rho_{h,o}$ is calculated as follows

$$\Delta\rho_{h,i} = (1 - f_{wi}) \cdot \rho_{headgroup} + f_{wi} \cdot \rho_0 - \rho_0 \quad (S15)$$

$$\Delta\rho_{h,o} = \left(1 - f_{w,o}\right) \cdot \rho_{headgroup} + f_{w,o} \cdot \rho_0 - \rho_0 \quad (S16)$$

where i=inner or o=outer headgroup, $\rho_{headgroup}$ is the scattering length density of the lipid headgroup, ρ_0 is the scattering length density of the water. The fraction of water in the inner and outer shell(30), f_w, is given by

$$f_{w,i} = 1 - \frac{V_{head}P_i}{((R_i + t_{shell})^3 - R_i^3)} \tag{S17}$$

$$f_{w,o} = 1 - \frac{V_{head}P_o}{(R_i + 2t_{shell} + D_c)^3 - (R_i + t_{shell} + D_c)^3} \tag{S18}$$

where P_i and P_o are the number of phospholipids of the inner and the outer leaflet of the vesicle respectively.

$$P_i = 4\pi \frac{(R_i + t_{shell} + D_c/2)^3 - (R_i + t_{shell})^3}{3V_{tail}} \tag{S19}$$

$$P_o = 4\pi \frac{(R_i + t_{shell} + D_c)^3 - (R_i + t_{shell} + D_c/2)^3}{3V_{tail}} \tag{S20}$$

where V_{tail} is the volume occupied by the hydrophobic tails of the phospholipid.

To extract k_{ex} and k_{flip} the excess fraction of either H- and D-lipid in the inner and outer and leaflet as a function of time can be analysed using:

$$f_{out}(t) = \left(\frac{k_{ex} - Z}{Y - Z}\right) \exp\left(-Y(t-d)\right) + \left(\frac{Y - k_{ex}}{Y - Z}\right) \exp\left(-Z(t-d)\right) \tag{S21}$$

$$f_{in}(t) = \left(\frac{k_{ex} - Z}{Y - Z}\right) \cdot \frac{k_{flip} + k_{ex} - Y}{k_{flip}} \exp\left(-Y(t-d)\right) + \left(\frac{Y - k_{ex}}{Y - Z}\right) \cdot \frac{k_{flip} + k_{ex} - Z}{k_{flip}} \exp\left(-Z(t-d)\right) \tag{S22}$$

where d is a delay time.

Four-concentric shell model results:

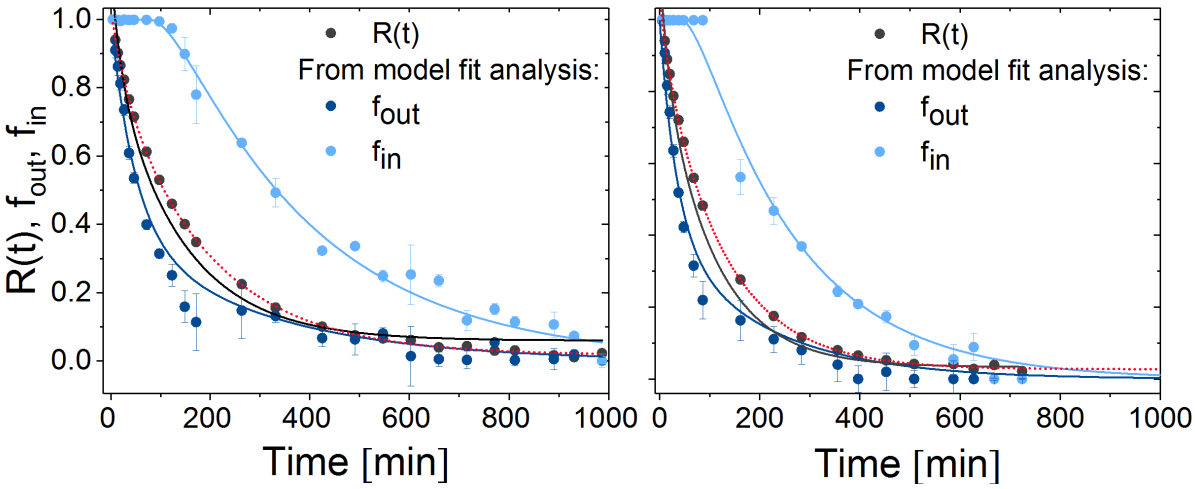

Figure S1: Results for exchange and flip-flop of liposomes without peptide (A) and with peptide (B). The plot shows the excess fraction of either H- and D-lipid in the inner and outer and leaflet ($f_{in}(t)$ and $f_{out}(t)$ respectively) based on direct modelling of full Q range TR-SANS data, and the R(t) curves extracted from the integral net loss of scattering intensity (Eq. 1). The solid black line is a result of a joint fit of all curves extracting information on the exchange (k_{ex}) and flip-flop (k_{flip}) rates, while the red dotted line represents a separate fit to only the R(t) curve.

Structural fit parameter of liposomes using the 3-shell model:

Table S1: Structural fit parameter of liposomes with and without indolicidin (1:20) at different temperatures, as based on SANS data.

	**27 ° C**		**37 ° C**		**47 ° C**	
	**No peptide**	**peptide**	**No peptide**	**peptide**	**No peptide**	**peptide**
**R_i [nm]**	40	38.5-44[d]	40	39-54[d]	40	41.7-62[d]
**V_{head}[Å^3]**[a]	330	330	332	332	334	334
**V_{tail}[Å^3]**[a]	770	770	775	775	785	785
**t_{shell} [Å]**[b]	6	6	6	6	6	6
**D_c [Å]**[b]	26	26	25	25	24	24
**f_{PEG}**[c]	0.025	0.04	0.025	0.04	0.025	0.05
**Rg_{PEG} [Å]**[b]	15	15	15	15	15	15

[a]*Hard constrained parameters*[43-44]. [b]*Soft constrained by limits.* [c]*Allowed to vary slightly in liposome samples with peptide to account for the extra protiated peptide material (the value is based on a fit of the end state curve).* [d]*Parameter dependent on time, see Figure 4.*

Concentration dependence:

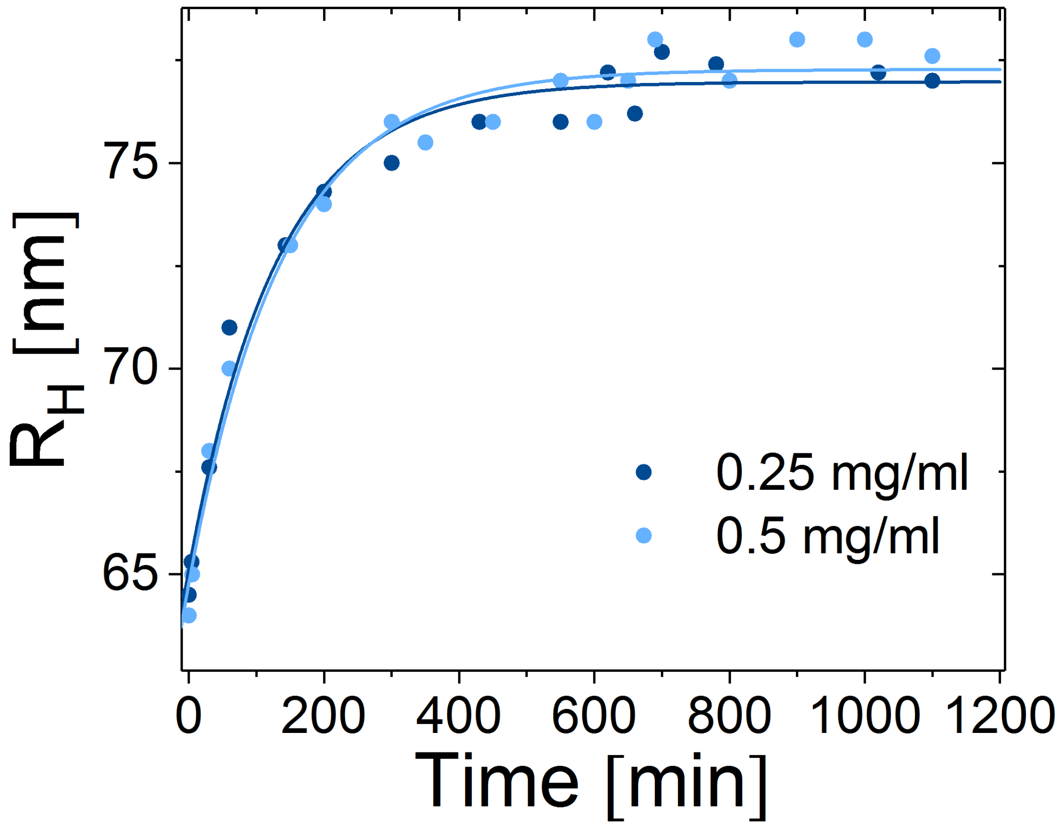

Figure S2: Comparing the hydrodynamic radius of liposomes with 1:20 indolicidin over time, at an overall lipid concentration of 0.25 mg/ml and 0.5 mg/ml as seen by dynamic light scattering.

The effect of pre-incubation of liposomes with peptide versus freshly mixing:

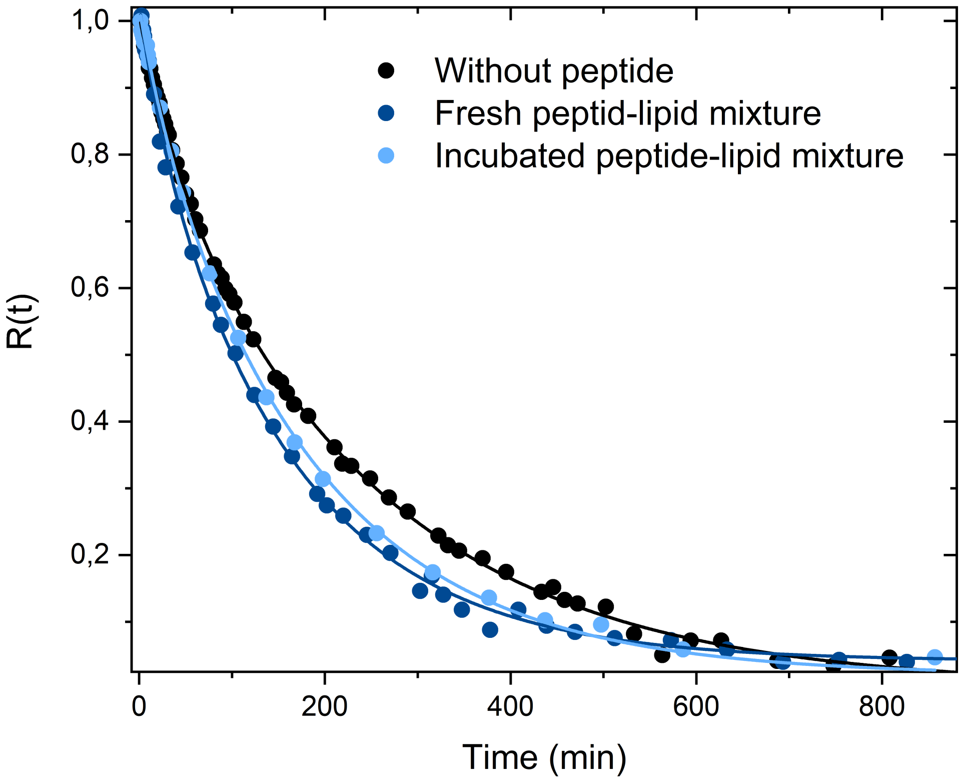

Figure S3: TR-SANS data comparing pre-incubating DMPC/DMPG liposomes with 1:20 indolicidin with freshly mixing the liposome and peptide solution at time=0. Experiment conducted at 37 °C on the D22 beamline at ILL.

Table S2: Thermodynamical parameters on TR-SANS data presented in Figure S3.

	k_{ex}* $[min^{-1}]$	k_{flip}* $[min^{-1}]$
Without peptide	$1.7\cdot10^{-2}\pm2.1\cdot10^{-4}$	$7.1\cdot10^{-3}\pm3.6\cdot10^{-4}$
Fresh peptide-lipid mixture	$2.4\cdot10^{-2}\pm7.0\cdot10^{-4}$	$1.2\cdot10^{-2}\pm1.2\cdot10^{-3}$
Incubated peptide-lipid mixture	$1.9\cdot10^{-2}\pm5.0\cdot10^{-4}$	$1.1\cdot10^{-2}\pm9.9\cdot10^{-4}$

**The rate constants (k) of exchange and "flip-flop" are extrapolated to 37.0 °C from the Arrhenius data.*

Comparing exchange and flip-flop rates of DMPC and DMPG lipids in vesicles:

Table S3: Thermodynamical parameters on TR-SANS data presented in Figure 8.

	k_{ex}* $[min^{-1}]$	k_{flip}* $[min^{-1}]$
	**No peptide**	
DMPC/DMPG visible**	$1.8 \cdot 10^{-2} \pm 3.4 \cdot 10^{-4}$	$7.3 \cdot 10^{-3} \pm 4.3 \cdot 10^{-4}$
DMPC visible	$9.1 \cdot 10^{-3} \pm 1.1 \cdot 10^{-3}$	$6.8 \cdot 10^{-3} \pm 1.1 \cdot 10^{-3}$
	**With added peptide**	
DMPC/DMPG visible**	$3.2 \cdot 10^{-2} \pm 7.0 \cdot 10^{-4}$	$1.5 \cdot 10^{-2} \pm 1.4 \cdot 10^{-3}$
DMPC visible	$1.6 \cdot 10^{-2} \pm 5.2 \cdot 10^{-4}$	$7.1 \cdot 10^{-2} \pm 1.3 \cdot 10^{-3}$

**The rate constants (k) of exchange and "flip-flop" are extrapolated to 37.0 °C from the Arrhenius data.*

*** Rates also presented in Table 3, but repeated here for easier comparison.*
